# Multifractal analysis of heart rate variability in pregnancy during sleep

**DOI:** 10.3389/fcvm.2024.1404055

**Published:** 2024-08-06

**Authors:** Martin O. Mendez, Anna M. Bianchi, Florian Recker, Brigitte Strizek, J. S. Murguía, Pierluigi Reali, Jorge Jimenez-Cruz

**Affiliations:** ^1^ Department of Obstetrics and Prenatal Medicine, University of Bonn, Bonn, Germany; ^2^ Department of Electronics, Information and Bioengineering, Politecnico di Milano, Milan, Italy; ^3^ Science Faculty, Universidad Autonoma de San Luis Potosi, San Luis Potosi, Mexico

**Keywords:** cardiovascular system, autonomic nervous system, detrended fluctuation analysis, prenatal medicine, sleep

## Abstract

Understanding the complex dynamics of heart rate variability (HRV) during pregnancy is crucial for monitoring both maternal well-being and fetal health. In this study, we use the Multifractal Detrended Fluctuations Analysis approach to investigate HRV patterns in pregnant individuals during sleep based on RR interval maxima (MM fluctuations). In addition, we study the type of multifractality within MM fluctuations, that is, if it arises from a broad probability density function or from varying long-range correlations. Furthermore, to provide a comprehensive view of HRV changes during sleep in pregnancy, classical temporal and spectral HRV indices were calculated at quarterly intervals during sleep. Our study population consists of 21 recordings from nonpregnant women, 18 from the first trimester (early-pregnancy) and 18 from the second trimester (middle-pregnancy) of pregnancy. Results. There are statistically significant differences (p-value < 0.05) in mean heart rate, rms heart rate, mean MM fluctuations, and standard deviation of MM fluctuations, particularly in the third and fourth quarter of sleep between pregnant and non-pregnant states. In addition, the early-pregnancy group shows significant differences (p-value < 0.05) in spectral indices during the first and fourth quarter of sleep compared to the non-pregnancy group. Furthermore, the results of our research show striking similarities in the average multifractal structure of MM fluctuations between pregnant and non-pregnant states during normal sleep. These results highlight the influence of different long-range correlations within the MM fluctuations, which could be primarily associated with the emergence of sleep cycles on multifractality during sleep. Finally, we performed a separability analysis between groups using temporal and spectral HRV indices as features per sleep quarter. Employing only three features after Principal Component Analysis (PCA) to the original feature set, achieving complete separability among all groups appears feasible. Using multifractal analysis, our study provides a comprehensive understanding of the complex HRV patterns during pregnancy, which holds promise for maternal and fetal health monitoring. The separability analysis also provides valuable insights into the potential for group differentiation using simple measures such as mean heart rate, rms heart rate, and mean MM fluctuations or in the transformed feature space based on PCA.

## 1 Introduction

During pregnancy, the autonomic nervous system (ANS) undergoes significant changes to accommodate the physiological demands of the mother and support the developing fetus. The ANS is responsible for regulating various involuntary processes in the body, such as heart rate, blood pressure, digestion, and respiration (1). For instance, some of these changes are that heart rate tends to increase gradually during pregnancy, mainly due to the influence of hormonal changes and increased blood volume. Blood pressure also undergoes alterations, with a decrease in systemic vascular resistance and a subsequent reduction in blood pressure during the first two trimesters, followed by a gradual return to pre-pregnancy levels in the third trimester. One of the key adaptations in the ANS during pregnancy is an overall increase in sympathetic activity (2). This shift in autonomic balance helps to create an environment conducive to fetal growth and development. In addition, other systems related to the ANS, such as the respiratory system, are also affected during pregnancy, leading to higher minute ventilation (the total amount of air inhaled and exhaled per minute) (3). However, certain pregnancy complications, such as hypertensive disorders of pregnancy (HDP) or gestational diabetes mellitus (GDM), can disrupt the normal autonomic regulation, generating complications in both maternal and fetal health (4–7).

Heart rate variability (HRV) refers to the variation in time intervals between consecutive heartbeats (8), and it is considered a valuable measure of the ANS activity (9). Self-affinity, in the context of HRV, refers to the presence of statistical similarity or scaling properties across different time scales within the HRV signal. When HRV exhibits self-affinity, it means that the fluctuations in the time intervals between heartbeats display similar patterns or characteristics at different time scales. This property implies that the HRV signal possesses a certain level of complexity and organization (10, 11). For example, healthy individuals often exhibit statistical similarity patterns in their HRV, indicating a balanced and adaptable autonomic control of the heart rate. Reduced or altered self-affinity may indicate impaired autonomic regulation and increased risk of cardiovascular diseases, such as hypertension, heart failure, and arrhythmias. It can also be indicative of physiological stress, inflammation, or other disturbances in the body (12–15). The self-affinity analysis of HRV is commonly performed using techniques such as detrended fluctuation analysis (DFA) or fractal analysis (16). These methods quantify the presence and degree of self-affinity by examining how the fluctuations in HRV persist or decay across different time scales. Self-affinity in HRV has been studied in the context of pregnancy to understand the changes in ANS regulation in short-time recordings (17, 18), which suggest changes in the fractal-like behavior and complexity of HRV patterns during different stages of pregnancy. In general, the scaling exponents of HRV increase as gestation time advances, while the HRV complexity decreases (19). However, the reason is unclear since it may be influenced by hormonal changes, increased sympathetic activity, and other physiological adjustments related to pregnancy, given fetal development and growth.

As mentioned above, there are studies characterizing changes in HRV during pregnancy and the postpartum period, but they have been limited to short-term recordings of electrocardiographic (ECG) signals during wakefulness. Few studies have analyzed classic temporal and frequency indices of HRV during sleep at different stages of pregnancy (8, 20). However, sleep deserves more attention because it is a fundamental process that plays an essential role in maintaining overall health and well-being, and its importance is heightened during pregnancy. Sleep acts as a catalyst for immune function, facilitates tissue repair, and increases overall vitality. In addition, the emotional benefits of proper sleep are remarkable, acting as a shield against stress, anxiety, and mood swings - factors that are particularly prevalent during pregnancy (21). The effects of sleep extend to the growth and development of the fetus, as the body releases essential hormones that play a key role in the proper development of the fetus. An often underestimated aspect of sleep is its role in maintaining healthy blood pressure levels. This is particularly important during pregnancy when maintaining optimal blood pressure is essential to prevent complications such as pre-eclampsia. Sleep also plays a role in managing blood sugar levels and promoting healthy weight gain. Finally, sleep is a complex process involving many sub-processes during the different sleep stages, so it would be useful to establish indices to characterize it. Therefore, understanding the relationship between HRV, self-affinity, pregnancy, and sleep may provide insights into maternal cardiovascular health and sleep-related complications during this crucial period.

Sleep is a cyclical process characterized by multiple stages that influence the oscillatory components of HRV. In particular, the high-frequency (between 0.15 Hz and 0.5 Hz) and low-frequency (between 0.04 Hz and 0.15 Hz) components contribute to the self-affinity of cardiac activity, which is often described by a monofractal structure with 1/f behavior (15, 22). Additionally, some studies have shown that HRV also shows some characteristics of multifractality during wakefulness at different pathologies (23, 24), in 24h recordings (25), during sleep (26, 27) and in different stages of sleep (28). However, during sleep, the dominance of the high-frequency component could obscure valuable insights into the multifractality of cardiovascular dynamics during sleep, since slow and small variations with valuable information could be hidden by this oscillatory rhythm (29, 30). To address this issue and accurately characterize a possible multifractal self-affinity inherent to the sleep process, it would be interesting to assess variations in HRV amplitude after mitigating the impact of high-frequency components.

Thus, we are interested in analyzing sleep HRV patterns during pregnancy using classical and Multifractal indices to better understand the dynamics of the ANS regulation during sleep in different physiological conditions (early-pregnancy, middle-pregnancy, and non-pregnancy). The main aim is a better understanding of the underlying mechanisms: How does the autonomic nervous system controlling heart rate is modified during the different stages of pregnancy and compared to non-pregnancy?

The objective of this research is to study the self-affinity patterns exhibited by the ANS throughout the HRV indices during sleep in three distinct women groups: non-pregnancy, early-pregnancy, and middle-pregnancy. The analysis of self-affinity behavior is carried out through the widely recognized Multifractal Detrended Fluctuation Analysis methodology. Additionally, the sleep period is segmented into four intervals, and classical temporal and spectral indexes of heart rate variability are computed and subsequently compared across these segments. Lastly, we have analyzed the original feature space of the HRV indexes and its transformed version using Principal Component Analysis (PCA) with the aim of highlighting differences between non-pregnancy, early-pregnancy, and middle-pregnancy individuals.

## 2 Methodology

**Figure 1:**
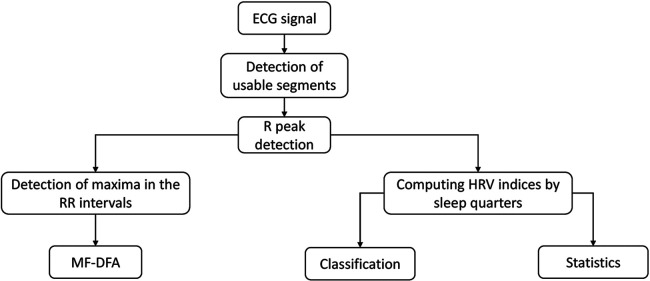
Schematic representation of the methodology process.

Figure 1 illustrates the procedural workflow used in this study. The process begins with the ECG signal, in which specific segments relevant to our analysis were identified and selected. This is followed by a precise detection of the R peaks. From this point, the process is divided into two distinct analyses. The first analysis focuses on the multifractal assessment, which is based on the detection of maxima within the RR intervals. In parallel, the second analysis focuses on the more traditional HRV analysis together with maxima within the RR intervals, including classification (class separation) and statistical evaluations.

### 2.1 Polysomnography data

Twenty-one whole-night polysomnographies (PSG) from Healthy (*n* = 6) and Nocturnal Frontal Lobe Epilepsy (NFLE, *n* = 15) women in reproductive age were downloaded from the CAP database. The database consists of 108 PSG recordings in EDF format acquired at the Sleep Disorders Center of the Ospedale Maggiore of Parma, Italy. This database is freely available from Physionet (http://www.physionet.org). The sleep recordings present at least three electroencephalography (EEG) channels, electrooculography (EOG), electromyography (EMG) of the submentalis muscle, bilateral anterior tibial EMG, respiratory signal, and ECG (sampling frequencies varied between 100 Hz and 512 Hz). The sleep stages were scored by expert neurologists following the gold standard rules (31). The complete database comprises the following groups: healthy (*n* = 16), nocturnal frontal lobe epilepsy (*n* = 40), REM Behavior Disorder (*n* = 22), periodic leg movement (*n* = 10), insomniac (*n* = 9), narcoleptic (*n* = 5), sleep disorder breathing (*n* = 4), and bruxism (*n* = 2). We selected recordings of women from the healthy and NFLE groups since the sleep profile was similar and the HRV was not altered by pathological events such as apneas. Besides, this similarity between healthy and NFLE subjects has been previously documented in the literature (32).

Eighteen whole-night PSGs of early-pregnant and eighteen ones of middle-pregnant nulliparous women were downloaded from the National Sleep Research Resource (NSRR) repository (funded by the National Heart, Lung, and Blood Institute from USA). The recordings belong to the study, called The Nulliparous Pregnancy Outcomes Study: Monitoring Mothers-to-Be (nuMoM2b) (33, 34). The goal of the database was to provide valuable information to healthcare professionals and women who are pregnant or planning to become pregnant and to provide data and knowledge for future research. Most participants underwent two PSGs, the first between weeks 6 and 15 (3072 records for the first trimester), and the second between weeks 22 and 29 (2805 records for the second trimester). At each of the two recordings, data were collected through clinical measurements, personal interviews, and self-administered questionnaires. Participants ranged in age from 14 to 44 years. Each PSG has airflow (AF), snoring, nasal pressure, abdominal and thoracic effort, blood oxygen saturation (SpO2), and ECG (sampled at 200 Hz). Each PSG comes with an XML file with wake-sleep annotation and the time of occurrence of events: central apnea, obstructive apnea, hypopnea, SpO2 desaturation, and noise in SpO2. For the signal acquisition the Embletta Gold digital recorder was used and the technical details can be found in the manual of operation (33, 34). Table 1 shows the demographic information of the women who were recorded. Information is presented as median and min-max interval. NFLE and healthy recordings are shown as non-pregnancy.

**Table 1:** Median and interval of the demographic information of the women making up the study groups.

	Non-pregnancy (*N* = 21)	Early-pregnancy (*N* = 18)	Middle-pregnancy (*N* = 18)
Age (yr)	30.0 [16.0, 42.0]	25.5 [18.0, 32.0]	26.5 [19, 32]
BMI (kg/$m^{2}$)	–	26.7 [21.0, 39.1]	29.3.0 [22, 41.5]
Desaturation time (s)	–	52.0 [4.0, 405.0]	115.00 [101.0, 265.0]
Apnea (quantity)	–	0.00 [0.0, 2.0]	0.00 [0.0, 5.0]
Useful record (h)	7.5 [5.3, 9.2]	7.00 [4.5, 10.5]	7.4 [4, 9.8]

BMI, Body mass index.

It is important to note that the data come from two different data sets, which could cause inhomogeneity in the data. However, the acquisition protocol follows the standards accepted for clinical and home monitoring of sleep, with sampling frequencies for the ECG sufficient to correctly detect the R-peaks (between 100 and 512 Hz). In addition, it is worth mentioning that the HRV signal extraction only depends on the identification of the R peak on the ECG, which is possible even in presence of noise or on different ECG leads, thus it presents the advantage of being one of the physiological signals more robust to the noise and device independent in different data sets. In addition we tested and compared the HRV metrics from the healthy and NFLE groups through the Mann-Whitney U test and Brown-Forsythe test: no one of the HRV metrics resulted different between the two groups confirming their homogeneity.

As a final note regarding the non-pregnant group, despite a thorough search of a database of sleep recordings from women, we did not find a free database with healthy, nulliparous, young women to match the nuMoM2b dataset. To overcome this situation for future research, we are in the process of formulating a protocol to acquire recordings for a control group, with the goal of complementing the nuMoM2b dataset.

### 2.2 Pre-processing of the ECG signal

The pre-processing stage helps to improve the quality of the ECG signal, reduces noise and artifacts, and prepares the signal for further analysis, such as HRV assessment or feature extraction for clinical diagnosis. However, motion artifacts contaminating the ECG signal and missing segments of ECG are common in sleep recordings, so their identification is necessary before any other procedure.

#### 2.2.1 Non-useful segments and R-peak detection

From each PSG, the ECG signal was extracted and used to locate the inter-beat time by detecting the R-peaks. R-peak detection is an important step since self-affinity analysis will be based on the occurrence of R-peaks. Therefore, it is necessary to define the ECG segments in which R-peaks can be reliably detected.

The amplitude of the ECG signal depends on the subject and varies throughout the night due to body movements caused by changes in body position, apneas, etc. Therefore, it is necessary to normalize the ECG to mitigate the effect of ECG amplitude change on the development of non-useful segment detectors. For this purpose, non-overlapping 10 s segments of the ECG signal were normalized with respect to their variance. Then, to detect signal segments with motion artifacts (non-useful segments), the trend was removed from each segment, and its variance was calculated. If the variance was between a value of 0.04 and 0.2 (note that segments were previously normalized with respect to the variance), the segment was considered ECG; otherwise, it was annotated as a non-useful segment. The optimal threshold interval was based on the analysis of manually annotated segments from ten polysomnographic sleep ECG signals. For all the recordings, the first and last 20 min were considered non-useful segments.

We then used two different approaches for automatic R-peak detection: the widely recognized Pan-Tompkins algorithm (35) and a custom procedure based on wavelet decomposition. This dual-method strategy was adopted because of the variance in signal characteristics, which warranted the use of the most effective technique for detecting accurate R-peaks in each scenario.

Following this step, we proceeded to eliminate non-useful segments and wake epochs from the R-peak series. The segments remaining after filtering were then merged. It’s worth noting that this process has minimal impact on the assessment of the scaling behavior (36). To ensure the robustness of our analysis, we only considered recordings with a minimum duration of 5 h for the calculation of self-affinity, in line with maintaining at least 3 sleep NREM-REM cycles.

#### 2.2.2 Heart rate variability fluctuations

After obtaining the R-peaks series from the previous step, the consecutive R-peak intervals, often referred to as RR intervals or inter-beat intervals, were calculated. These RR intervals were then subjected to cubic spline interpolation to create a regularly spaced time series with a 4 Hz sampling rate.

The RR intervals exhibit fluctuation around a mean value, representing the mean Heart Rate. For extended time periods like sleep, these RR intervals tend to form a Gaussian distribution. However, for shorter time spans, variations in the properties of the global Gaussian distribution can be observed. These variations might correlate with sleep stages or sequences of pathological events. Therefore, an appropriate method is needed to account for these local variations over time. Multifractal Detrended Fluctuation Analysis (MF-DFA) is particularly well-suited for this purpose because it extends beyond the limitations of the standard DFA by considering the scaling of qth-order moments at different time scales (37, 38). Unlike standard DFA, which specifically focuses on the second moment (q=2), MF-DFA considers different qth-order moments, providing a more comprehensive perspective on the multifractal nature of the data. The latter approach allows for identifying or detecting intricate correlations and patterns that may remain unnoticed using other methods (27).

However, during sleep, the prevalence of parasympathetic activity, combined with the significant effect of breathing on the cardiovascular system, enhances the oscillatory rhythm within the RR intervals. This rhythm contains remarkable information on the HRV dynamics, but could also prevent some techniques from revealing other interesting features in the RR intervals (29, 30). Therefore, a time series was constructed to counterbalance the influence of this oscillatory rhythm. This series includes only the maxima of the RR intervals (here called MM fluctuations). This time series could also be extracted with various analytical methods, such as the Hilbert transform (29), empirical mode decomposition, wavelets, filtering techniques, or adaptive filtering.

**Figure 2:**
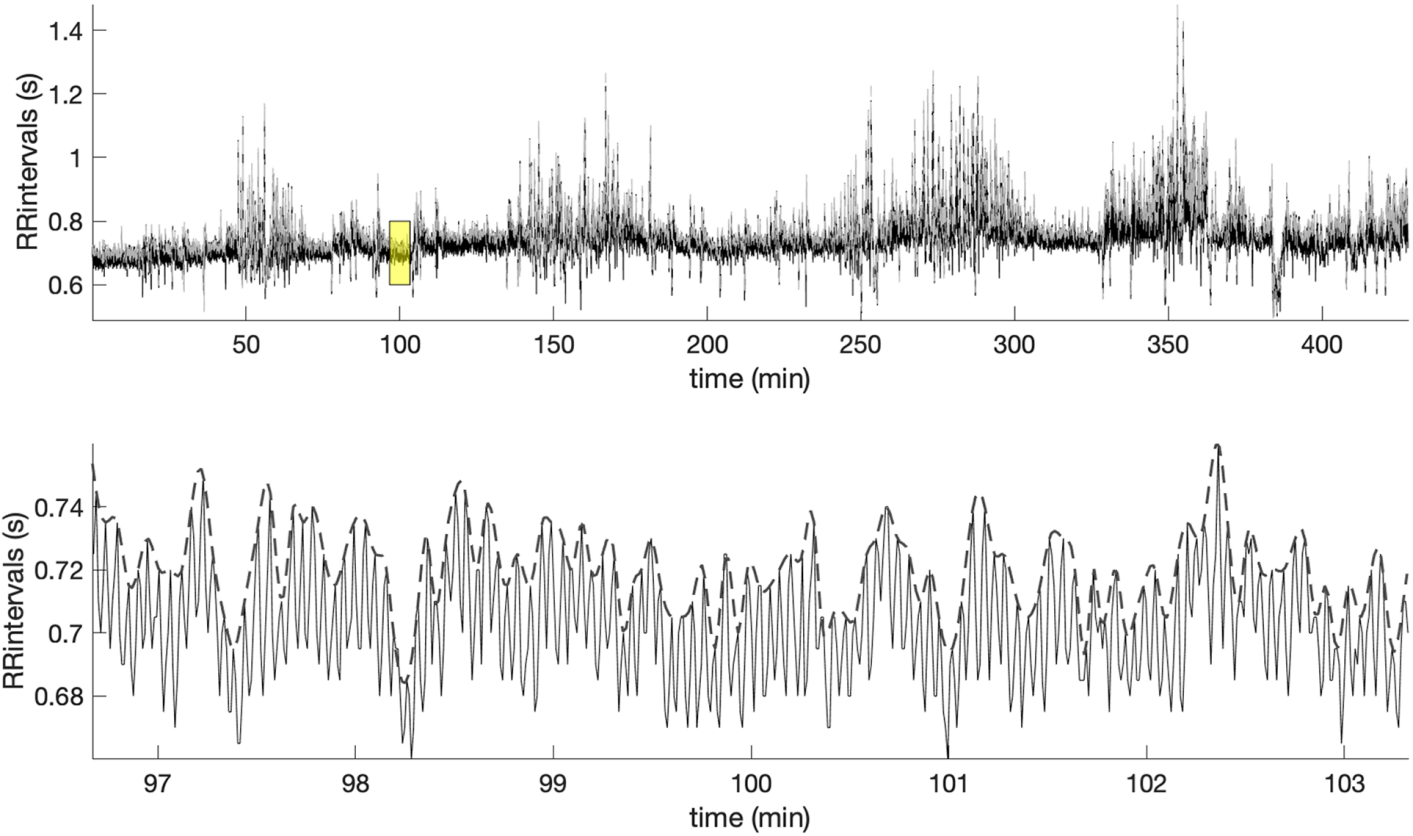
Example of the RR intervals during sleep and along with the superimposed MM fluctuations (gray line). The lower panel offers a zoomed-in view of the highlighted yellow window, showing the MM fluctuations (dashed line).

Figure 2 shows in the upper panel the RR intervals (black line) during the sleep of an early-pregnancy record and the overlapping amplitude of the MM fluctuations (gray line). As we can easily see, the MM fluctuations contain information related to the slow oscillations of the RR intervals. A zoom of the yellow window is shown in the lower panel, where the MM fluctuations are depicted with a dashed line. MM fluctuations were used to assess the multifractal characteristics of the cardiovascular system during the sleep phase using MF-DFA.

### 2.3 Multifractal detrended fluctuation analysis (MF-DFA)

MF-DFA is a technique used to assess the presence of fractal scaling behavior in physiological signals such as HRV. The MF-DFA method involves the following steps:

1. Obtain a time series: The time series can be a sequence of evenly spaced time points, n, representing measurements over time, x[n].
2. Calculate the cumulative sum of the time series: This step involves integrating the time series minus the mean time series,
  (1) $$y[n]=\sum_{k=1}^{n}(x[k]-E(x[n])),\;n=1,\ldots ,N,$$
  where N is the number of data points and E(x[n]) is the mean.
3. Divide the cumulative sum, y[n], into non-overlapping segments: The time series is divided into smaller segments of equal length. $N_{s}=[N/s]$, segment of length s.
4. Fit a polynomial function to each segment: A polynomial function, typically a straight line, is fitted to each segment of the cumulative sum, $y_{s}[n])$.
5. Calculate the q-th order fluctuation function: The q-th order deviations of the data points from the corresponding polynomial fit are calculated for each segment,
  (2) $$F_{q}(s)={\left(\frac{1}{N}\sum_{n=1}^{N}(y[n]-y_{s}[n]{)}^{q}\right)}^{\frac{1}{q}},\;q\in R$$

If the time series under analysis have fractal properties, the $F_{q}(s)$ should follow a power-law of the form:

(3) $$F_{q}(s)∼s^{H(q)},$$

where H(q) can be conceptualized as an extension of the Hurst exponent, with the equivalence H(2)≡α of the DFA exponent. If H(q) remains constant regardless of changes in q, the studied time series exhibits monofractal characteristics. Conversely, if H(q) varies with q, indicating a diverse scaling behavior, the time series possesses multifractal attributes. When q assumes positive values, H(q) represents the scaling behavior of the segments with large fluctuations. Conversely, for negative q values, H(q) characterizes the scaling dynamics of segments with small fluctuations.

The conventional approach to present the results of MF-DFA involves a two-step conversion process. First, H(q) is transformed into the q-order mass exponent (τ(q)), and subsequently, τ(q) is further transformed into the q-order singularity exponent (h) and the q-order singularity dimension (D(h)) (37, 38). This process finishes in the creation of a graphical representation known as the multifractal spectrum, where D(h) is plotted against h. This is:

(4) τ(q)=qH(q)−1,

(5) $$h=\frac{d\tau (q)}{dq},$$

(6) D(h)=qh−τ(q).

### 2.4 Time and frequency domain indices of HRV

We calculated temporal and frequency indices of HRV to establish a benchmark in line with current research standards. These indices serve as important reference points, as there is a well-established link between these indices and the function of the parasympathetic and sympathetic branches of the ANS. The sleep time was partitioned into four non-overlapping segments, named Q1, Q2, Q3 and Q4. This segmentation was done to effectively capture the variations that occur during different moments of sleep. We adopted this strategy in order to overcome the usual sleep staging based on EEG and provide parameters able to quantify sleep characteristics in a simpler way along the night. We then calculated the indices separately for each of these segments.

*Time domain indices:*

• meanRR, representing the average of the RR intervals.
• sdRR, which measures the deviation of the RR intervals with respect to meanRR.
• rmsRR, which calculates the root mean square of the time differences between consecutive normal heartbeats.
• meanMM, which represents the average MM fluctuation; it can be correlated with respiratory behavior.
• sdMM, which measures the deviation of the MM fluctuations with respect to meanMM.

*Frequency domain indices:*

The Power Spectral Density (PSD) was computed using the Welch method. Specifically, the signal was segmented into eight parts with a 50% overlap, and each segment was windowed using a Hamming window. The periodograms of these segments were then averaged to obtain the final PSD estimate, from which the following indices were calculated.

• Low frequency (LF), the power within the frequency band from 0.04 Hz to 0.15 Hz.
• High frequency (HF), the power within the frequency band from 0.15 Hz to 0.5 Hz; it is particularly associated with parasympathetic activity and respiration.
• LF/HF ratio, a traditional indicator of sympathovagal balance that provides insight into the balance between sympathetic and parasympathetic activity. However, the context of the recording must be considered for proper interpretation and, in particular the possible shift of the HF component towards the LF frequency range, may lead to erroneous interpretation; On the other hand, the LF/HF ratio was frequently used in sleep studies where the respiration frequency is more regular making the interpretation more robust (39, 40).

To ensure a meaningful comparison between different sleep segments and participants, we normalized the power of the PSD that belongs to the HF and LF bands with respect to the power of both the low and high-frequency bands. In essence, the summation of the power in the low and high-frequency bands approaches unity.

### 2.5 Multifractal analysis of the MM fluctuations

The multifractal spectrum was derived from the MM fluctuations of the RR intervals. This was done using the Multifractal Detrended Fluctuation Analysis (MF-DFA) method with linear detrending. The analysis included a spectrum of scales ranging from 16 to 2,048 and involved the evaluation of different q-orders, specifically [−5,−3,−1,0,1,3,5]. From the multifractal spectrum, we extracted the multifractal indices, including the position $\arg\max_{h}D(h)$ and the width of D(h) shown in Figure 4.

Generating surrogate data is a common technique used in time series analysis to test hypotheses and assess the significance of observed patterns by comparing them to randomized versions of the original data. There are several methods (random shuffle, phase randomization, iterative amplitude adjusted Fourier transform, etc.) for generating surrogate data, each designed to preserve certain characteristics of the original time series while destroying the ones to be tested. In this study, the random shuffle method was chosen, which consists of randomly shuffling the original data points to destroy any temporal correlation in the signal while preserving the distribution of the values (mean value, variance). Thus, this is useful to evaluate the presence of temporal structure or autocorrelation in the data, both in the short and in the long time scales. In general, the procedure involves: (a) taking the original time series, and (b) randomly permute the order of the data points. This process could be repeated many times to generate multiple surrogate data sets. When the surrogated data show lower correlation with respect to the original one we may conclude that measured correlations are a feature characterizing the signal.

Thus, to assess the type of multifractality, we calculated surrogate MM fluctuations. This involved analyzing the MM fluctuations that were computed from the same RR intervals but in different order. In the shuffling procedure, the RR interval values were randomly reordered, which effectively destroyed all correlations related to the sleep stages. The MM fluctuations were then calculated and resampled at 4 Hz.

### 2.6 Statistics and classification

We calculated temporal and spectral indices of HRV within different sleep segments, each corresponding to one-quarter of the total sleep duration (designated Q1, Q2, Q3, and Q4). We then performed a two-way mixed Analysis Of Variance (ANOVA) for each HRV index, considering PREGNANCY as a three-level between-subject factor (non-pregnancy, early-pregnancy, and middle-pregnancy) and SLEEPQ as a within-subject factor with four levels (Q1, Q2, Q3, and Q4). For each index, we first analyzed the interaction PREGNANCY*SLEEPQ; if the interaction was found significant, we analyzed simple main effects. Conversely, in the absence of a significant interaction, the main effects of PREGNANCY and SLEEPQ were evaluated separately. In case of significant differences from the above evaluations, pairwise multiple comparisons (i.e., post-hoc tests) were made using the Bonferroni correction to account for the increasing type-I error due to multiple testing. The effect of PREGNANCY was also analyzed on the multifractal indices derived from the MM fluctuations by means of a one-way ANOVA, followed by the appropriate Bonferroni-corrected post hoc analysis in case significant differences were found. The analyzed data distributions were checked for approximate normality and homogeneity of variance using the Shapiro-Wilk and Levene tests, respectively. The sphericity assumption for the mixed ANOVAs was tested through the Mauchly test; if not met, the Greenhouse-Geisser correction was applied to the degrees of freedom of the within-subject analysis. A p-value less than 0.05 was considered statistically significant for every analysis. The presence of outliers that may introduce severe bias in the results was established by inspecting boxplots of data grouped by PREGNANCY and SLEEPQ. In general, subjects were considered outliers if their data points appeared distant from the range $\left[Q_{\mathrm{low}}-1.5\cdot IQR,Q_{\mathrm{up}}+1.5\cdot IQR\right]$, with IQR, $Q_{\mathrm{low}}$, and $Q_{\mathrm{up}}$ indicating the interquartile range, the lower, and upper quartiles, respectively. Only the most distant outliers were excluded from the analysis, and no more than one subject per group of PREGNANCY (i.e., less than 6% of the original data). The statistical analysis was carried out in SPSS Statistics (IBM, U.S.A.).

#### 2.6.1 Classification

In the field of biomedical engineering, it is of great importance to determine whether information derived from physiological signals contributes to the automatic detection of medical conditions. Given the pressing need to explore novel features that can improve the classification process across different states in women, it is useful to investigate whether HRV indices, even in the absence of sleep stage annotations, can serve as discriminative features.

To understand this, we evaluated the separability among groups by plotting the following indices from the different sleep quarters:

(a) three of the HRV indices that were statistically different among the three groups.
(b) the first three scores obtained from the PCA applied to the whole set of HRV indices.

PCA is a method used for dimensionality reduction. The primary objective is to transform the original variables into a new set of variables, the principal components, which are orthogonal and capture the maximum variance in the data.

Given a dataset X consisting of n observations and p variables, the steps involved in PCA are:

1. Compute the Covariance Matrix:
  (7) $$\Sigma =\frac{1}{n-1}(X-\bar{X}{)}^{T}(X-\bar{X})$$
  Here, $\bar{X}$ is the mean-centered data matrix.
2. Eigenvalue Decomposition: Solve the eigenvalue problem for Σ:
  (8) Σv=λv
  Where λ are the eigenvalues and v are the corresponding eigenvectors.
3. Order and Select Principal Components: Arrange the eigenvalues in descending order and choose the first k eigenvectors corresponding to the largest k eigenvalues to represent the k principal components.
4. Compute Scores: The scores represent the projection of the original data onto the principal components. For the $i^{\mathrm{th}}$ observation $x_{i}$, the scores $z_{\mathrm{ij}}$ for the $j^{\mathrm{th}}$ principal component is given by:
  (9) $$z_{\mathrm{ij}}=x_{i}^{T}v_{j}$$
  Here, $v_{j}$ is the $j^{\mathrm{th}}$ eigenvector.
5. Transform Data: Transform the original data X into the new coordinate system using the selected principal components:
  (10) $$Y=XV_{k}$$
  Where $V_{k}$ contains the first k eigenvectors.

In this context, the scores provide a representation of each observation in terms of the principal components. They capture the essential information from the original data in a reduced dimensionality space.

## 3 Results

This section presents the results of the statistical analysis conducted on heart rate fluctuations across the three PREGNANCY groups and the four sleep quarters (SLEEPQ). These results include the multifractal analysis combined with an examination of the surrogate MM time series. Subsequently, classical HRV indices and their temporal evolution during sleep are presented. Finally, the separability of the three groups in the original feature space of the HRV indices and after PCA is investigated.

### 3.1 Multifractal analysis of MM fluctuations

**Figure 3:**
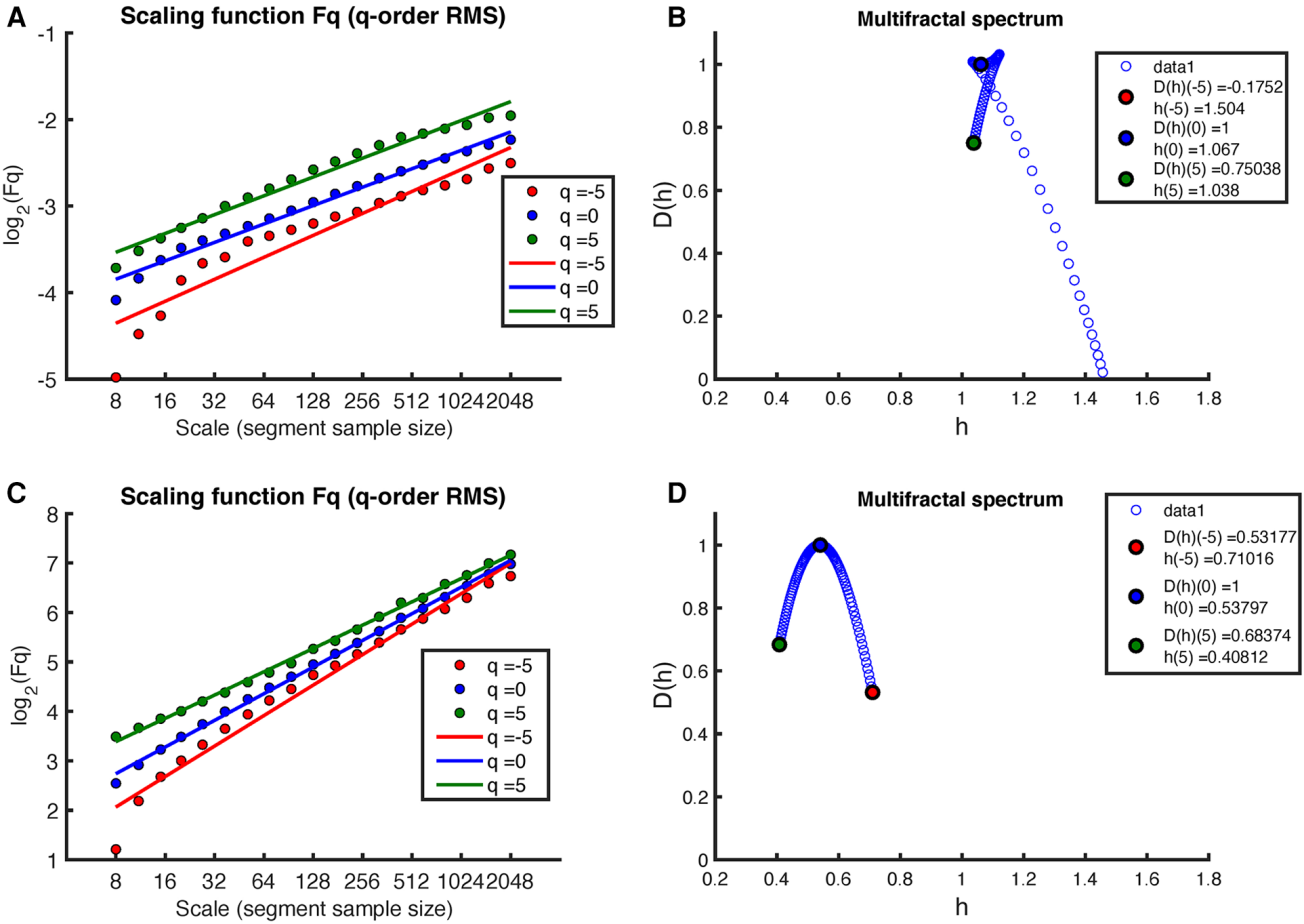
Example of MF-DFA applied to RR intervals during sleep in an early-pregnancy recording. The figure includes plots of $F_{q}(s)$ for different q-orders RMS and the corresponding regression for both (**A**) the original RR intervals and (**C**) the shuffled RR intervals. (**B**) shows the corresponding multifractal spectrum for the original RR intervals, while (**D**) shows the multifractal spectrum for the shuffled RR intervals.

Figure 3 provides a representative illustration of the results obtained by applying the MF-DFA method, with q-orders ={−5,0,5} root-mean-square (RMS), to the RR intervals extracted from a full night recording of an early-pregnant subject (please see Figure 2 for the original RR intervals). These results (Figures 3A,B) are contrasted with those obtained after shuffling the same RR intervals (Figures 3C,D). When the actual sequence of RR intervals is considered, the regression lines of the different q-orders show a similar slope (Figure 3A, time series with a structure close to 1/f noise), which does not allow observing the multifractality of time series. Thus, as shown in Figure 3B, the multifractal spectrum is concentrated within a small interval of h close to one. Figure 3C shows the regression lines of the MF-DFA of the shuffled RR intervals also at different q-orders ={−5,0,5} RMS. Again, all slopes of the regression lines are close to h≈0.5, indicating a random white noise. The Figure 3D shows multifractal spectrum small D(h) width with singular exponents h concentrated around 0.5 and multifractal dimension close to 1.

**Figure 4:**
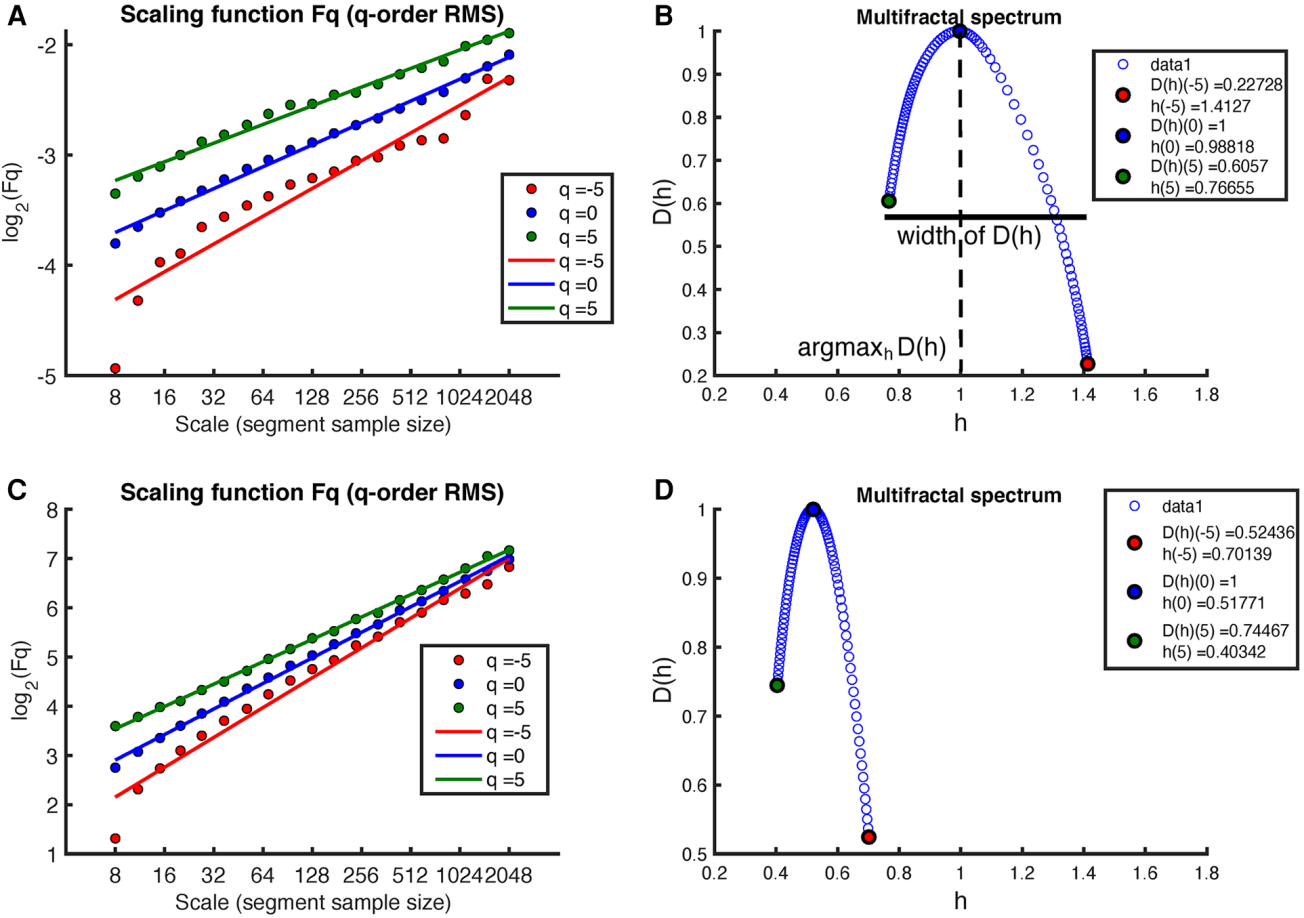
Example of MF-DFA applied to MM intervals during sleep in an early-pregnancy recording. The figure includes plots of $F_{q}(s)$ for different q-orders RMS and the corresponding regression for both (**A**) the MM fluctuations and (**C**) the surrogate MM fluctuations. (**B**) shows the corresponding multifractal spectrum for the MM fluctuations, while (**D**) shows the multifractal spectrum for the surrogate MM fluctuations.

Figure 4 shows the results of applying the MF-DFA method to the MM fluctuations obtained from the same sleep recording (see Figure 2 for the original MM fluctuations). Again, the MF-DFA was performed for q-orders ={−5,0,5} RMS, and the results are shown in Figure 4A. This time, the regression lines of the different q-orders do not show a similar slope, which implies that the scaling behavior is different depending on the q-order, around the 1/f noise. The multifractal spectrum spans over a wide interval of h (see Figure 4B). On the other hand, the regression lines of the MF-DFA of the surrogate MM intervals are shown in Figure 3C. The slopes of these regression lines are around h≈0.5, indicating that the surrogate series tends to have a random white noise behavior. As can be seen in Figure 3D, the multifractal spectrum is centered at 0.5, but the long-term correlations are not completely destroyed.

Table 2 shows the mean and standard deviation of the indices computed from the multifractal spectrum, including the width of D(h) and $\arg\max_{h}D(h)$, grouped by PREGNANCY. The Hurst exponent H(q=2), as defined by the monofractal DFA, characterizes the overall fractal structure of the time series and correlates with $\arg\max_{h}D(h)$, which represents the central tendency within the multifractal spectrum. The width of the multifractal spectrum, on the other hand, represents the deviation from the average fractal structure for segments with both large and small fluctuations. Both the width and central tendency of the multifractal spectrum provide important insights into the characteristics of the time series and can help to understand changes within the time series. For the groups under study (non-pregnancy, early-pregnancy, middle-pregnancy), the calculated D(h) widths and $\arg\max_{h}D(h)$ were found approximately normally distributed and homoscedastic. The effect of PREGNANCY was found not significant for both D(h) width ($F(2,54)=0.149,p=0.862,partial\;\eta^{2}=0.005$) and $\arg\max_{h}D(h)$ ($F(2,54)=1.346,p=0.269,partial\;\eta^{2}=0.047$), meaning no statistically significant changes among groups were observed in the MM fluctuations during sleep.

**Table 2:** Mean and standard deviation of the indices extracted from the multifractal spectrum D(h). No statistically significant differences were observed among groups.

Index	Non-pregnancy	Early-pregnancy	Middle-pregnancy
$\arg\max_{h}D(h)$	1.07 ± 0.04	1.06 ± 0.03	1.08 ± 0.05
Width D(h)	0.78 ± 0.13	0.77 ± 0.18	0.80 ± 0.14

**Figure 5:**
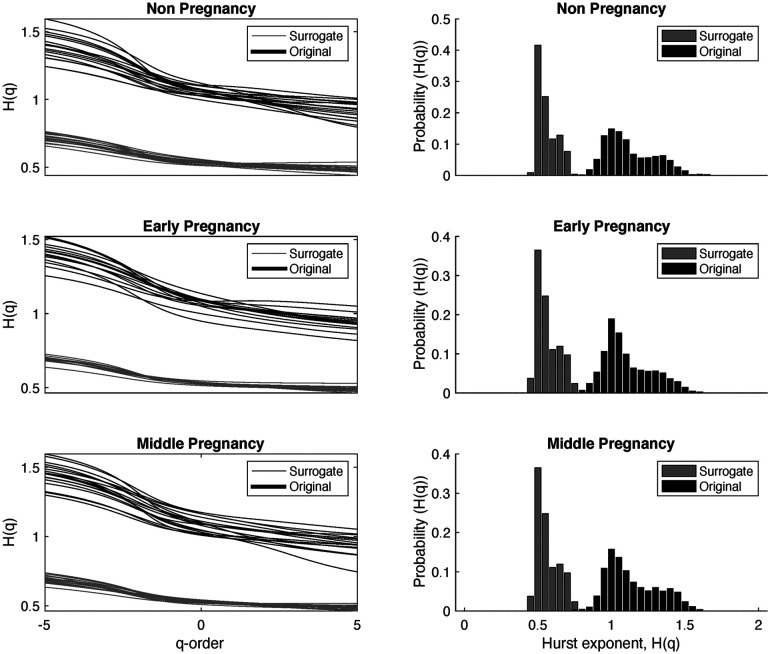
Behavior of the Hurts exponent of the MM fluctuations at different q-order RMS, obtained from the RR series and the shuffled RR series, shown by groups. The black color represents the results of the MM fluctuations computed from the original RR intervals, while the grey color represents the results of the MM fluctuations computed from the shuffled RR intervals. The first column shows the Hurts exponent for different values of q-orders, and the second column shows the probability distribution of the Hurts exponent independent of q.

Figure 5 illustrates the behavior of the Hurts exponent for MM fluctuations at various q-order RMS values. These values are derived from both RR series and shuffled RR series, organized by group: non-pregnancy, early-pregnancy, and middle-pregnancy. The Hurts exponent of MM fluctuations is shown in a distinctive background color (Original), while the corresponding values from surrogate MM fluctuations are shown in gray (Surrogate).

The main objective here is to identify the underlying multifractality nature of the time series data. If $H_{\mathrm{Surrogate}}(q)=0.5$, this suggests that the surrogate time series comes from a time series with multifractality due to different long-range correlations. Conversely, when $H_{\mathrm{Original}}(q)=H_{\mathrm{Surrogate}}(q)$, it implies that the independence between samples remains unchanged and the multifractality arises from the probability density.

The first column in Figure 5 shows H(q) values against different q-order RMS. The second column shows the probability of occurrence for different H(q) values, independent of q. In particular, $H_{\mathrm{Original}}(q)$ has values roughly between 1.5 (for negative q=−5) and 0.8 (for positive q=5). The peak in the probability density of $H_{\mathrm{Original}}(q)$ is observed around $H_{\mathrm{Original}}(q)\approx 1$, suggesting that most of the curves have similar values of $H_{\mathrm{Original}}(q=2)$.

When analyzing the behavior of $H_{\mathrm{Surrogate}}(q)$, a similar trend to $H_{\mathrm{Original}}(q)$ is observed, but with values approximately in the range of 0.7 (for negative q) and 0.4 (for positive q). The maximum in the probability density of $H_{\mathrm{Surrogate}}(q)$ occurs around $H_{\mathrm{Original}}(q)\approx 0.5$, indicating a white noise structure independent of the q-order. Furthermore, there is less dispersion in the $H_{\mathrm{Surrogate}}(q)$ values. This behavior is consistent for all three groups. Statistical analysis was carried out and showed significant differences between $H_{\mathrm{Original}}(q)$ and $H_{\mathrm{Surrogate}}(q)$ at each q-order, (p-value less than 0.05).

### 3.2 Analysis of the HRV indices

Table 3 shows the results of the HRV indices calculated by sleep time quarter (Q1, Q2, Q3, and Q4) for the non-, early- and middle-pregnancy groups. To ensure normality of the distributions and homogeneity of variance across the three groups of PREGNANCY for each SLEEPQ level, extreme outliers were identified using the approach described in Section 2.6. Specifically, three subjects, one for each PREGNANCY group, were excluded from the analysis because they showed outliers for some frequency-domain HRV indices. Therefore, the number of subjects included was 20 for the non-pregnancy, 17 for the early-pregnancy, and 17 for the middle-pregnancy group.

**Table 3:** Mean and standard deviation (SD) of the MM and RR indices reported for all combinations of SLEEPQ (Q1, Q2, Q3, Q4) and PREGNANCY (non-pregnancy, *N* = 20; early-pregnancy, *N* = 17; middle-pregnancy, *N* = 17) levels. Values are reported as mean ± SD. Pairwise differences are indicated for indices reporting a significant interaction SLEEPQ*PREGNANCY.

Index/time	Non-pregnancy				Early-pregnancy			
	Q1	Q2	Q3	Q4	Q1	Q2	Q3	Q4
meanMM (s)	4.02 ± 0.35	4.06 ± 0.34	4.14 ± 0.37	4.19 ± 0.37	3.67 ± 0.41	3.71 ± 0.36	3.76 ± 0.39	3.83 ± 0.36
sdMM ($s^{2}$)	1.21 ± 0.17	1.33 ± 0.22	1.45 ± 0.27	1.49 ± 0.29	0.94 ± 0.12	1.08 ± 0.14	1.10 ± 0.12	1.12 ± 0.14
meanRR (s)	0.90 ± 0.10^a,b,d^	0.93 ± 0.10^a,b,d^	0.97 ± 0.11^c,d^	0.99 ± 0.12^c,d^	0.83 ± 0.12^a,b^	0.84 ± 0.11^a,b^	0.87 ± 0.12	0.89 ± 0.13
sdRR ($s^{2}$)	0.072 ± 0.022	0.075 ± 0.021	0.087 ± 0.028	0.095 ± 0.030	0.062 ± 0.023	0.074 ± 0.021	0.079 ± 0.024	0.088 ± 0.031
rmsRR (s)	0.044 ± 0.017	0.046 ± 0.019	0.054 ± 0.023	0.056 ± 0.021	0.046 ± 0.024	0.045 ± 0.021	0.050 ± 0.023	0.057 ± 0.025
HF (nu)	0.64 ± 0.10	0.63 ± 0.10	0.63 ± 0.11	0.61 ± 0.12	0.76 ± 0.07	0.71 ± 0.05	0.69 ± 0.06	0.71 ± 0.07
LF (nu)	0.36 ± 0.10	0.37 ± 0.10	0.37 ± 0.11	0.39 ± 0.12	0.24 ± 0.07	0.29 ± 0.05	0.31 ± 0.06	0.29 ± 0.07
LF/HF	0.60 ± 0.24	0.63 ± 0.33	0.64 ± 0.30	0.69 ± 0.34	0.33 ± 0.13	0.42 ± 0.11	0.46 ± 0.14	0.43 ± 0.14
α	0.89 ± 0.06	0.91 ± 0.06	0.93 ± 0.04	0.94 ± 0.05	0.87 ± 0.07	0.95 ± 0.04	0.93 ± 0.04	0.94 ± 0.05
	Middle-pregnancy							
Index/time	Q1	Q2	Q3	Q4				
meanMM (s)	3.72 ± 0.41	3.74 ± 0.43	3.81 ± 0.39	3.86 ± 0.41				
sdMM (s)	0.97 ± 0.20	1.09 ± 0.18	1.16 ± 0.22	1.19 ± 0.21				
meanRR (s)	0.78 ± 0.13	0.79 ± 0.12	0.79 ± 0.11	0.80 ± 0.10				
sdRR (s)	0.056 ± 0.025	0.059 ± 0.022	0.063 ± 0.021	0.067 ± 0.022				
rmsRR (s)	0.035 ± 0.019	0.034 ± 0.019	0.034 ± 0.014	0.037 ± 0.017				
HF (nu)	0.74 ± 0.08	0.70 ± 0.09	0.69 ± 0.11	0.70 ± 0.09				
LF (nu)	0.26 ± 0.08	0.30 ± 0.09	0.31 ± 0.11	0.30 ± 0.09				
LF/HF	0.36 ± 0.15	0.44 ± 0.18	0.49 ± 0.26	0.45 ± 0.19				
α	0.92 ± 0.06	0.94 ± 0.06	0.95 ± 0.07	0.97 ± 0.09				

^a^Significantly different from Q3 sleep quarter within the same pregnancy group, p< 0.05.

^b^Significantly different from Q4 sleep quarter within the same pregnancy group, p< 0.05.

^c^Significantly different from early-pregnancy in the same sleep quarter, p< 0.05.

^d^Significantly different from middle-pregnancy in the same sleep quarter, p< 0.05.

The interaction PREGNANCY*SLEEPQ was significant only for meanRR (*F*(4.474, 114.083) = 4.032, *p* = 0.003, $partial\;\eta^{2}=0.137$), with the subsequent post hoc analysis of simple main effects pointing out significant differences between non-pregnant and middle-pregnant women in every sleep time quarter (Q1: p=0.005, Q2: p=0.001, Q3: p<0.001, Q4: p<0.001) and between non-pregnant and early-pregnant subjects in Q3 (p=0.027) and Q4 (p=0.038). In both cases, non-pregnant women showed significantly increased meanRR compared to early- and middle-pregnant ones. Pairwise significant differences were also detected between sleep time quarters within the non-pregnancy (Q1–Q3: p<0.001, Q1–Q4: p<0.001, Q2–Q3: p<0.001, Q2–Q4: p<0.001) and early-pregnancy groups (Q1–Q3: p=0.035, Q1–Q4: p=0.003, Q2–Q3: p=0.005, Q2–Q4: p=0.002), but not within the middle-pregnancy one suggesting that heart rate changes during the night are reduced in advanced pregnancy stages. As for the other HRV indices reported in Table 3, no statistically significant interactions were found between pregnancy groups and sleep time quarters. Therefore, only the main effects of PREGNANCY and SLEEPQ are analyzed in the following, with the relevant Estimated Marginal Means (EMM) reported in Table 4. sdRR revealed significant main effects of both PREGNANCY ($F(2,51)=4.132,p=0.022,partial\;\eta^{2}=0.139$) and SLEEPQ ($F(2.218,113.116)=28.748,p<0.001,partial\;\eta^{2}=0.360$). Specifically, significant differences were found between the EMMs of the non-pregnancy and middle-pregnancy groups (p=0.020) and between the EMMs of all sleep time quarters (Q1–Q2: p=0.013, Q1–Q3: p<0.001, Q1–Q4: p<0.001, Q2–Q3: p<0.001, Q2–Q4: p<0.001, Q3–Q4: p=0.010). In particular, the sdRR of the non-pregnant subjects was significantly higher compared to the middle-pregnant and was found to significantly increase over time during sleep.

**Table 4:** Estimated Marginal Means (EMMs) and model standard error (SE) for the evaluation of SLEEPQ (Q1, Q2, Q3, Q4) and PREGNANCY (non-pregnancy, *N* = 20; early-pregnancy, *N* = 17; middle-pregnancy, *N* = 17) main effects on the MM and RR fluctuations. Values are reported as EMM ± SE. Pairwise differences are indicated for indices reporting significant main effects. Blank lines mark indices that showed a significant interaction SLEEPQ*PREGNANCY, requiring simple main effects to be evaluated instead (see Table 3).

Index	EMMs by SLEEPQ				EMMs by PREGNANCY		
	Q1	Q2	Q3	Q4	Non-pregnancy	Early-pregnancy	Middle-pregnancy
meanMM (s)	3.80 ± 0.05^b,c^	3.84 ± 0.05^b,c^	3.90 ± 0.05	3.96 ± 0.05	4.10 ± 0.08^d,e^	3.74 ± 0.09	3.78 ± 0.09
sdMM (s)	1.04 ± 0.02^a,b,c^	1.17 ± 0.03^b,c^	1.24 ± 0.03	1.26 ± 0.03	1.37 ± 0.04^d,e^	1.06 ± 0.04	1.10 ± 0.04
meanRR (s)	–	–	–	–	–	–	–
sdRR (s)	0.063 ± 0.003^a,b,c^	0.069 ± 0.003^b,c^	0.076 ± 0.003^c^	0.083 ± 0.004	0.082 ± 0.005^e^	0.076 ± 0.005	0.061 ± 0.005
rmsRR (s)	0.042 ± 0.003^c^	0.042 ± 0.003^b,c^	0.046 ± 0.003^c^	0.050 ± 0.003	0.050 ± 0.004	0.050 ± 0.005	0.035 ± 0.005
HF (nu)	0.72 ± 0.01^b^	0.68 ± 0.01	0.67 ± 0.01	0.67 ± 0.01	0.63 ± 0.02^d,e^	0.72 ± 0.02	0.71 ± 0.02
LF (nu)	0.29 ± 0.01^b^	0.32 ± 0.01	0.33 ± 0.01	0.33 ± 0.01	0.37 ± 0.02^d,e^	0.28 ± 0.02	0.29 ± 0.02
LF/HF	0.43 ± 0.03^b^	0.50 ± 0.03	0.53 ± 0.03	0.52 ± 0.03	0.64 ± 0.04^d,e^	0.41 ± 0.04	0.44 ± 0.04
α	0.90 ± 0.01^a,b,c^	0.94 ± 0.01	0.94 ± 0.01	0.95 ± 0.01	0.92 ± 0.01	0.92 ± 0.01	0.95 ± 0.01

^a^Significantly different from Q2 sleep quarter, p< 0.05.

^b^Significantly different from Q3 sleep quarter, p< 0.05.

^c^Significantly different from Q4 sleep quarter, p< 0.05.

^d^Significantly different from early-pregnancy, p< 0.05.

^e^Significantly different from middle-pregnancy, p< 0.05.

rmsRR showed significant main effects of both PREGNANCY ($F(2,51)=3.380,p=0.042,partial\;\eta^{2}=0.117$) and SLEEPQ ($F(2.100,107.084)=11.014,p<0.001,partial\;\eta^{2}=0.178$), but significant pairwise differences were found only between sleep time quarters (Q1–Q4: p=0.002, Q2–Q3: p=0.006, Q2–Q4: p<0.001, Q3–Q4: p=0.010). In particular, rmsRR was significantly higher during the last sleep time quarter (Q4) compared to previous ones, and during Q3 with respect to Q2.

Since the LF and HF powers were evaluated in normalized units (n.u.), the same statistical results apply to both. Specifically, these features showed significant main effects of both PREGNANCY ($F(2,51)=8.283,p<0.001,partial\;\eta^{2}=0.245$) and SLEEPQ ($F(2.548,129.946)=5.704,p=0.002,partial\;\eta^{2}=0.101$), with the non-pregnancy group showing significant pairwise differences compared to both the early-pregnancy (p=0.002) and middle-pregnancy (p=0.005) ones. Specifically, HF power was significantly lower in the non-pregnant subjects compared to the early- and middle-pregnant. Besides, a significant difference emerged between Q1 and Q3 sleep time quarters (p=0.008), with Q1 reporting significantly higher HF power than Q3. Due to the above normalization, LF powers showed the same effects as HF but in the opposite direction.

LF/HF revealed significant main effects of both PREGNANCY ($F(2,51)=9.668,p<0.001,partial\;\eta^{2}=0.275$) and SLEEPQ ($F(3,153)=4.044,p=0.008,partial\;\eta^{2}=0.073$). The post hoc analyses pointed out significant pairwise differences between non-pregnant and both early- (p<0.001) and middle-pregnant (p=0.003) subjects, as well as significant differences between the Q1 and Q3 sleep time quarters (p=0.022). Specifically, the LF/HF of the non-pregnant women was higher than that of early- and middle-pregnant ones, and Q3 showed a significant increase in this index compared to Q1.

As for the α calculated with the DFA, only the main effect of SLEEPQ was found significant (*F*(2.379, 121.317) = 13.907, *p* < 0.001, $partial\;\eta^{2}=0.214$), with the post hoc analysis showing a significant increase of α over sleep time quarters compared to Q1 (Q1–Q2: p<0.001, Q1–Q3: p<0.001, Q1–Q4: p<0.001).

MM fluctuations reported statistically significant differences in both meanMM and sdMM indices, showing significant main effects of PREGNANCY (meanMM: *F*(2, 51) = 5.560, *p* = 0.007, $partial\;\eta^{2}=0.179$, sdMM: *F*(2, 51) = 19.391, *p* < 0.001, $partial\;\eta^{2}=0.432$) and SLEEPQ (meanMM: *F*(2.583, 131.728) = 16.024, *p* < 0.001, $partial\;\eta^{2}=0.239$, sdMM: *F*(2.385, 121.658) = 32.716, *p* < 0.001, $partial\;\eta^{2}=0.391$). The subsequent pairwise comparisons highlighted statistically significant increases in meanMM and sdMM in non-pregnant subjects compared to both early- (meanMM: p=0.012, sdMM: p<0.001) and middle-pregnant (meanMM: p=0.031, sdMM: p<0.001) ones. Besides, these indices showed significant differences across pairs of sleep time quarters, with both distinguishing Q1 from Q3 (meanMM: p=0.003, sdMM: p<0.001) and Q4 (meanMM: p<0.001, sdMM: p<0.001) and Q2 from Q3 (meanMM: p=0.008, sdMM: p=0.002) and Q4 (meanMM: p<0.001, sdMM: p=0.006). Specifically, both meanMM and sdMM were found to increase during Q3 and Q4 compared to Q1. In addition, sdMM showed a significant increase in Q2 compared to Q1 (p<0.001). meanMM and sdMM could be correlated with respiratory behavior since they are extracted from the maxima of the RR intervals, where the high frequency is influenced by the respiratory cycle. In particular, sdMM is expected to increase throughout the night because more time is generally spent in REM, where oscillatory breathing is known to be less stable. This fact explains the significant differences we observed between the first two sleep time quarters and the next ones.

### 3.3 Class separation

**Figure 6:**
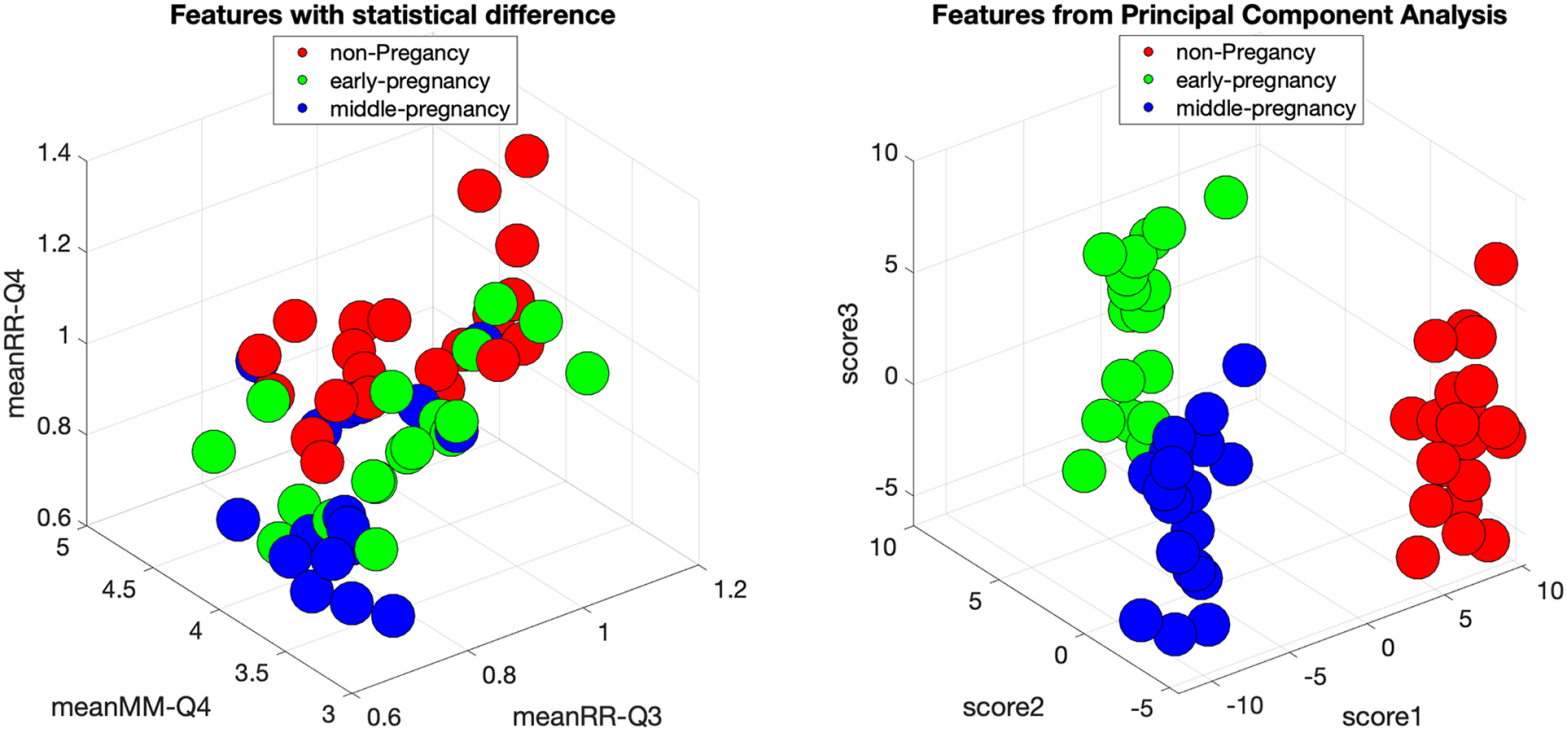
Feature space of original HRV indices with statistical significance and feature space of HRV indices transformed by PCA for the groups of non-pregnancy, early-pregnancy, and middle-pregnancy. The circles are the individual subjects with red color for non-pregnancy, green color for early-pregnancy, and blue color for middle-pregnancy.

Figure 6 shows the scatter plot for three HRV indices with statistical significance, together with the scatter plot of the three features with the highest explanatory value after PCA transformation of the original feature set. Each feature was normalized with respect to its variance. Each data point represents an individual subject and is color-coded for clarity: the non-pregnant group is labeled red, the early-pregnant group is presented in green, and the middle-pregnant group is shown in blue.

Upon examination, the original feature space shows a clear separation of the early-pregnancy group from the others. However, there is an overlap between the non-pregnant and middle-pregnant groups, suggesting a potential limitation in the classification process. To address this, it is useful to explore alternative feature combinations that may improve separability. Implementing a classification procedure coupled with feature selection may be a viable approach.

Interestingly, after applying PCA, the transformed feature space shows a clear distinction between all three groups. This suggests that a simple classification method, perhaps even a simple linear discriminant analysis or the use of simple thresholds, may be sufficient for effective classification in this transformed space.

## 4 Discussion

In our study, we investigated the dynamics of HRV during sleep at different stages of pregnancy and compared them to non-pregnant conditions. To this end, we calculated both traditional temporal and spectral HRV indices and investigated the multifractal characteristics of MM fluctuations. Our main findings can be summarised as follows: (1) The multifractal structure of MM fluctuations persist throughout pregnancy, (2) The multifractal characteristics of MM fluctuations are associated with long-term correlations generated by the sleep process, and (3) separating the three study groups (non-, early- and middle-pregnancy) based on HRV appears feasible.

Our results showed that cardiac autonomic regulation undergoes significant changes during normal pregnancy. In particular, the most significant changes occur during the early stages, with similar levels observed during middle-pregnancy. These findings are consistent with previous research by (8, 20, 41), where there are reported cardiovascular adaptations during early-pregnancy. In particular, our study parallels the observations of the latter study by assessing nocturnal heart rates at different stages of pregnancy. We found a significant decrease in RR intervals, which is consistent with the findings of the aforementioned research, but it is better expressed in the last sleep quarter of the sleep time.

From a different perspective, a reduction in the complexity of the HRV serves as an indicator of potential cardiovascular dysfunction, suggesting an increased risk of physiological and physical deterioration (13). While cardiovascular function is expected to adapt during pregnancy, leading to a reduction in HRV, it is important to recognize that this pattern may differ from the norm in complicated pregnancies (42, 43). This abnormality in HRV changes in complicated pregnancies could manifest in different properties of the HRV time series, such as mean value and regular or irregular patterns. In particular, autonomic abnormalities measured by HRV indices have been associated with pregnancies complicated by hypertensive disorders. In contrast, in patients with diabetes, assessments of autonomic dysfunction based on heart rate show no significant differences compared with healthy pregnancies (44). However, the potential for predicting clinical outcomes in pregnancy using HRV measurements warrants further investigation, especially during longer recordings in specific conditions such as sleep. It’s worth noting that previous studies have typically analyzed short segments of HRV (e.g., 5 min), which is sufficient to evaluate the chosen indices. However, monitoring HRV during sleep could provide a more comprehensive view of cardiovascular function and its relationship to different processes that typically occur during sleep, such as different sleep stages. This approach holds promise for a more holistic assessment of women’s health during pregnancy.

It is interesting to note that, within our study sample, the observed tendency of the HRV indices as a function of the sleep structure remains consistent across the different stages of pregnancy. In particular, the observed increases in the RR intervals, together with the associated variances and trends in HRV indices, show similarity between the non-, early- and middle-pregnancy groups. This remarkable consistency implies a parallel behaviour of the ANS during sleep, regardless of the specific stage of pregnancy. Furthermore, from a different perspective, our multifractal spectral analysis provides compelling evidence for the presence of comparable self-affinity given long-term correlations at different scales and power levels that are consistently observed across the different stages of pregnancy. These findings provide valuable insights into the enduring complexity of heart rate dynamics throughout pregnancy. They highlight the crucial role of multifractal analysis in elucidating the significance of long-term correlations within these dynamic fluctuations, thus providing a deeper insight into the intricate interplay of physiological processes during pregnancy. However, further analysis in diverse pathological populations may be required to provide greater clarity for a fuller understanding of the clinical applicability of our findings.

It is important to note that understanding the multifractality nature of the time series could also contribute to a better understanding of the source. Thus as described by Kantelhardt et al. (38), it is possible to categorize multifractality in time series into two different types:

1. Multifractality arising from a broad probability density function: In this scenario, multifractality arises from a broad probability density function associated with the values within the time series. Importantly, this form of multifractality cannot be eliminated by shuffling the series, as the underlying probability distribution remains unchanged.
2. Multifractality due to different long-range correlations: In contrast, this type of multifractality arises due to different long-range correlations exhibited by small and large fluctuations in the time series. Here, the probability density function governing the values may be regular and have finite moments, such as a Gaussian distribution. When the series is shuffled, all these long-range correlations are broken, resulting in non-multifractal scaling.

Our results show that the MM fluctuations exhibit multifractality arising from distinct long-range correlations. This type of multifractality is expected during regular sleep, as the emergence of sleep cycles and stages introduces specific patterns within the RR intervals. These patterns are potentially valuable for automated classifications of sleep stages, as demonstrated in previous studies (45). However, to gain a fuller understanding of MM fluctuations and their behaviour, further analysis is imperative, especially in the context of pathological conditions where the normal sleep structure is disrupted, potentially leading to changes in multifractality.

A limitation of our study is the relatively small number of records available for analysis. However, we anticipate that, in the coming years, we will be able to build up a more extensive database with a sufficient number of records at different stages of pregnancy and from non-pregnant women. This expansion will not only increase the robustness of our findings but will also facilitate a more comprehensive evaluation of the clinical applicability of our proposed methods. Whilst we have a greater volume of data from pregnant women, we are constrained by the limited number of non-pregnant data sets currently available for analysis. Therefore, we made a conscious decision to maintain a balanced representation by keeping a similar number of records for each group.

Another point worth acknowledging is the simplified annotation modality we adopted for sleep staging. Since in real-life scenarios, particularly for home monitoring applications, the aim is to minimize the number of sensors to ensure comfort and compliance with daily sleep monitoring routines, we decided to categorise sleep time into quarters, despite the possibility of creating a more granular sleep profile based on HRV indices with a segment resolution of five minutes or less. This decision is in line with practical considerations for real-world applications and a possible simplification of the sleep evaluation from a visualization point of view.

It is important to note that another option for analyzing the multifractality of HRV is by decomposing the signal into classical components (HF, LF, VLF) and analyzing each component individually (30). Techniques such as filtering, wavelets, and empirical mode decomposition could be used for this purpose. Furthermore, to understand multifractality at different scales, the multifractal-multiscale method could be used (26). Thus, there is room for improvement in analyzing and understanding the ANS mechanisms involved during sleep in pregnancy from a multifractal perspective.

Separability across different groups of pregnancy stages seems possible. However, as noted by Stein et al. (8), it is important to recognize that each woman starts with a unique cardiovascular baseline and each pregnancy represents a different physiological journey. Therefore, implementing a normalization process based on the non-pregnancy stage for each woman could potentially produce more robust classification results. Thus, a more effective approach is to assess individual-level changes in different HRV indices at different stages of pregnancy. This approach is particularly valuable because, although HRV typically decreases in the early stages of pregnancy, there is considerable individual variation in the magnitude and direction of these changes as pregnancy progresses. Much of our current knowledge of changes in cardiac autonomic regulation during pregnancy is based on pairwise comparisons between short-term or Holter-based heart rate data and single-point assessments of HRV during pregnancy, contrasted with non-pregnancy baseline values. Therefore, it is important to shift our perspective away from generalized metrics to a personalized, longitudinal approach that considers pairwise comparisons between successive stages of pregnancy. This approach allows for a comprehensive exploration of cardiovascular adaptability as a unique and personalized phenomenon, moving beyond generic benchmarks.

Finally, with the intention of answering the question posed in the introduction, we may say that the application of MF-DFA on MM fluctuations, along with time and frequency domain analyses of HRV during sleep provides new insights into the physiological changes that occur during pregnancy in several important ways: (a) Revealing Multifractal Characteristics: MF-DFA allows the detection of complex HRV patterns that are not apparent using traditional linear methods. This analysis can identify multifractality in the MM fluctuations, indicating multifractal characteristics of the regulation of the autonomic nervous system during pregnancy, which are dynamically modified along pregnancy; (b) Detailed temporal and spectral analysis: The computation of classical temporal and spectral HRV indices at quarterly intervals during sleep provides a dynamic view of HRV changes throughout the sleep. This temporal resolution captures the fluctuations in HRV due to different sleep cycles and provides insight into how pregnancy affects these patterns, and (c) Applicability: the calculated features are particularly suitable for continuous monitoring and may find application in the home evaluation of maternal health through the quantitative analysis of multifractal properties and significant HRV changes during pregnancy during sleep in the home setting. Understanding the distinct HRV patterns associated with different stages of pregnancy may lead to more personalized healthcare interventions tailored to the specific needs of pregnant individuals at different stages of pregnancy.

## 5 Conclusions

We investigated the dynamics of HRV during sleep at different stages of pregnancy and contrasted them with non-pregnant conditions. By calculating traditional temporal and spectral HRV indices and exploring the multifractal characteristics within the MM fluctuations, we observe significant changes in cardiac autonomic regulation during normal pregnancy, with the most pronounced changes occurring in early pregnancy, followed by stabilization in middle-pregnancy. We also observed a significant reduction in RR intervals, which was particularly pronounced in the last quarter of sleep. While such a decrease in HRV is expected during pregnancy due to adaptive cardiovascular changes, it is important to recognize that this pattern may deviate from the norm in complicated pregnancies. In addition, a consistency in sleep structure was observed across different stages of pregnancy, with similarities in MM fluctuations, variances, and HRV trends between non-pregnant, early-pregnant, and middle-pregnant groups. This finding suggests an overloading of the sleep function that produces similar behavior of the ANS during sleep, regardless of the specific stage of pregnancy. However, to gain a more complete understanding of MM fluctuations and their behavior, where normal sleep structure may be disrupted, further analysis is essential. The results contribute to a broader understanding of the health of women during this critical stage of life and highlight the importance of multifractal analysis and extended HRV monitoring during sleep in gynecological and obstetrical settings.

The main novelties of our study could be summarized as:

• Application of Multifractal Analysis to Pregnancy HRV: This study uniquely applies Multifractal Detrended Fluctuation Analysis to HRV patterns during pregnancy. Previous research has primarily focused on linear methods, which need proper signal segmentation in stationary fragments. This multifractal approach is innovative in the context of maternal health monitoring. The use of Multifractal Analysis allows a completely automatic estimation of parameters which are related to long-term dynamics, with no need of complex pre-processing or operator-dependent data segmentation. For this reason, the proposed method could be particularly suitable for monitoring through simple wearable devices and automatic analysis. In addition, it could provide a single index across each night, able to summarize the status of the woman, allowing simple comparisons and evaluations.
• Comprehensive temporal and spectral HRV analysis during sleep: By calculating classical temporal and spectral HRV indices at quarterly intervals during sleep, this research provides a dynamic understanding of HRV changes throughout the sleep time in pregnant women, a perspective that has not been extensively explored before.
• Separability analysis using PCA: The use of Principal Component Analysis to achieve separability between groups demonstrates the relevance of the modifications induced in the HRV controlling mechanisms by pregnancy. Our results demonstrate clear differences in comparison with non-pregnant women and between pregnancy stages (early- and middle-pregnancy).
• Implications for Pathology Identification: The findings of our study on HRV hold promise for identifying or characterizing pathologies related with pregnancy, such as diabetes or sleep apnea. Future studies will focus on these aspects, with potential diagnostic and therapeutical applications. This adds a significant level of novelty and practical relevance to the research.

## Data Availability

Publicly available datasets were analyzed in this study. This data can be found here: https://sleepdata.org/datasets/numom2b; https://archive.physionet.org/physiobank/database/capslpdb/.
